# Effect of A disintegrin and metalloproteinase 10 gene silencing on the proliferation, invasion and migration of the human tongue squamous cell carcinoma cell line TCA8113

**DOI:** 10.3892/mmr.2014.2717

**Published:** 2014-10-21

**Authors:** YUAN SHAO, XIAO-YING SHA, YAN-XIA BAI, FANG QUAN, SHENG-LI WU

**Affiliations:** 1 Department of Otorhinolaryngology, The First Affiliated Hospital of Xi’an Jiaotong University, Xi’an, Shaanxi 710061, P.R. China; 2 The Sixth Hepatic Disease Ward, The Affiliated Xi’an Eighth Hospital, Medical College of Xi’an Jiaotong University, Xi’an, Shaanxi 710061, P.R. China; 3 Department of Hepatobiliary Surgery, The First Affiliated Hospital of Xi’an Jiaotong University, Xi’an, Shaanxi 710061, P.R. China

**Keywords:** TCA8113, A disintegrin and metalloproteinase 10, proliferation, migration, invasion

## Abstract

The present study aimed to investigate the effect of A disintegrin and metalloproteinase 10 (ADAM10) gene silencing on the proliferation, migration and invasion of the human tongue squamous cell carcinoma cell line TCA8113. RNA interference was used to knock down the expression of ADAM10 in the TCA8113 cell line and the proliferation, migration and invasive ability of the treated cells were observed *in vitro*. The expression levels of epidermal growth factor receptor (EGFR) and E-cadherin in the treated cells were determined by western blot analysis. The proliferation, migration and invasion abilities of cells in the ADAM10 siRNA-treated group were significantly lower than those in the control groups (P<0.05). In addition, compared with the control groups, the expression levels of EGFR and E-cadherin in the ADAM10 siRNA-treated cells were significantly decreased (P<0.05) and increased (P<0.05), respectively. These results suggested that ADAM10 is important in regulating the proliferation, invasion and migration of the human tongue squamous cell carcinoma cell line TCA8113 and that the mechanism may, at least in part, be associated with the upregulation of EGFR and the downregulation of E-cadherin.

## Introduction

Oral cancer is the sixth most common type of cancer in humans and poses a significant global threat to public health. The five-year survival rate of oral cancer patients is ~50% overall (1). In addition, a previous study suggested an increase in this rate (2). The prognosis of patients with oral cancer is relatively poor, predominantly due to the high rate of local recurrence, even following curative surgical resection (3). The development and progression of oral cancer are promoted by alterations in multiple cellular signaling pathways (4). Establishing the molecular and cellular mechanisms involved in the pathogenesis of oral cancer may provide assistance in identifying novel predictive and prognostic biomarkers and in developing novel therapeutic targets to treat oral cancer.

The A disintegrin and metalloproteinase (ADAM) family is a class of type I transmembrane proteins that contain two main structural domains, the disintegrin domain and the matrix metalloproteinase domain (5). To date, >35 members of this family have been identified (6). Previous studies have demonstrated that members of the ADAM family are able to regulate the process of tumor metastasis in humans by degrading the extracellular matrix and controlling cell adhesion and movement (7,8). One member of the ADAM family, A disintegrin and metalloproteinase 10 (ADAM10), has been reported to act as a positive regulator of cancer progression in hepatocellular carcinoma (9), renal cell carcinoma (10), pancreatic carcinoma (11), lung cancer (12) and gastric carcinoma (13). It has been suggested that ADAM10 causes the shedding of various cell surface molecules, including cadherins and amyloid precursor protein (14). Through these actions, ADAM10 is able to modulate the tumor microenvironment and the key processes involved in the progression of cancer, including cell proliferation, migration and angiogenesis (15). However, few studies have investigated the correlation between ADAM10 and oral cancer (16). In the present study, the effect of ADAM10 gene silencing on the proliferation, migration and invasion of the human tongue squamous cell carcinoma cell line TCA8113 was investigated. In addition, the association between the expression of ADAM10 and the expression of epidermal growth factor receptor (EGFR) and E-cadherin proteins in TCA8113 cells was examined.

## Materials and methods

### Cell lines and cell culture

The human tongue squamous cell carcinoma cell line TCA8113 was obtained from the Center of Biomedical Experimental Research at the Medical School, Xi’an Jiaotong University (Xi’an, China) (17). TCA8113 cells were cultured in Dulbecco’s modified Eagle’s medium (Invitrogen Life Technologies, Carlsbad, CA, USA) containing 10% fetal bovine serum (FBS) and 1% penicillin/streptomycin (Invitrogen Life Technologies) and incubated at 37°C in an atmosphere containing 5% CO_2_.

### siRNA transfection

For the downregulation of the endogenous expression of ADAM10, the following siRNA duplex (Aoke Biological Technology Co., Ltd., Shanghai, China) was used: 5′-AGACAUUAUGAAGGAUUAUTT-3′. As a negative control, the unspecific scrambled siRNA duplex (5′-AGGUAGUGUA AUCGCCUUGTT-3′) was used.

The TCA8113 cells (1×10^5^) were seeded into six-well plates 24 h prior to transfection. Transfection of siRNA was performed using Lipofectamine 2000 (Invitrogen Life Technologies) and 10 nM siRNA duplex/well and the transfection procedure was performed as previously described (18). The experiment was repeated three times.

The present study comprised a total of four groups, the blank control group, the Lipo2000 group (cells treated with Lipofectamine 2000), the control siRNA group (Lipofectamine 2000 and negative control siRNA) and the ADAM10-siRNA group (cells were treated with Lipofectamine 2000 and ADAM10 siRNA).

### Reverse transcription quantitative polymerase chain reaction (RT-qPCR)

RT-qPCR analysis of the ADAM10 transcripts in the TCA8113 cells was performed using the PrimeScript RT reagent kit according to the manufacturer’s instructions (Takara Bio, Inc., Shiga, Japan). The ADAM10 gene was amplified by RT-qPCR using the specific primers; forward, 5′-CTGCCCAGCATCTGACCCTAA-3′ and reverse, 5′-TTGCCATCAGAACTGGCACAC-3′. Amplification specificity was further validated by melting curve analysis. GAPDH was used as an internal control. The RT-qPCR procedures were performed in triplicate and the data were analyzed using the comparative Ct method.

### Western blot analysis

The cells were washed with phosphate-buffered saline (PBS) twice and lysed on ice in a buffer containing 150 mM NaCl, 50 mM Tris-HCl (pH 7.4), 2 mM EDTA, 1% NP-40, 0.5% 3-[(3-cholamidopropyl)-dimethyl-ammonio]-1-propane sulfonate and 0.1% SDS, which were all purchased from Wuhan Boster Biological Technology, Ltd. (Wuhan, China). Electrophoresis on 10% polyacrylamide gels was performed using equal quantities of protein (20 μg/lane) from the cell lysates under non-reducing conditions. The proteins fractionated by SDS-PAGE were electrotransferred onto Immobilon-P membranes (Invitrogen Life Technologies). The membranes were inhibited with 5% non-fat skimmed milk and 0.1% Tween-20 in PBS for 2 h. Subsequently, the membrane was incubated for 2 h with rabbit anti-human ADAM10 (1:500; R&D Systems, Minneapolis, MN, USA), rabbit anti-human EGFR (1:1,000; Santa Cruz Biotechnology, Inc., Dallas, TX, USA), or rabbit anti-human E-cadherin (1:1,000, Santa Cruz Biotechnology, Inc.). Following washing, the proteins were visualized using an Easyblot enhanced chemiluminescence detection kit (Sangon Biotech, Inc., Shanghai, China) using the appropriate goat anti-rabbit immunoglobulin G horseradish peroxidase-conjugated secondary antibody (1:1,000; Amersham Pharmacia Biotech, Piscataway, NJ, USA). The rabbit anti-human β-actin antibody (1:1,000; Santa Cruz Biotechnology, Inc.) was used as an internal marker for control purposes.

### Proliferation assay

The MTT (Sigma, St. Louis, MO, USA) colorimetric assay was used to screen for cell proliferation. Briefly, the TCA8113 cells were seeded into eight 96-well plates at a density of 2×10^3^ cells/well and incubated in RPMI-1640 medium (Wuhan Boster Biological Technology, Ltd.) for 24, 48, 72 and 96 h following treatment, respectively. MTT (20 μl, 5 mg/ml) was added to each well and the cells were cultured for an additional 4 h. The formazan crystals were dissolved by addition of dimethylsulfoxide (Sigma) and the absorbance was determined using a Multiskan Spectrum microplate reader (Thermo Fisher, Waltham, MA, USA)at 490 nm. A growth curve was produced according to the optical density (OD) value alterations. The experiment was performed three times independently.

### Soft agar colony formation assay

The TCA8113 cells (4×10^3^) were mixed with 0.5% top agar and seeded into 24-well plates with 1% base agar (Wuhan Boster Biological Technology, Ltd.). These cells were then cultured in an incubator at 37°C for 10 days in 5% CO_2_ at 95% humidity. Subsequently, images of the cell colonies in the soft agar were captured and counted using a microscope (BX53, Olympus Corp., Tokyo, Japan). All the experiments were independently repeated at least three times.

### Cell migration assay

The effect of ADAM10 knockdown on TCA8113 cell migration was measured as the ability of cells to migrate through Transwell filters (6.5 mm diameter, 5 mm pore size; Haoran Bio-pharm Co., Ltd, Jiangxi, China). Transwell filters were coated with Matrigel (BD Biosciences, San Diego, CA, USA) for 1.5 h prior to adding the cells. The cells were detached using trypsin 24 h after ADAM10 siRNA transfection. The cells were then seeded into the upper chamber of the Transwell (1×10^5^ cells/well) to enable migration to the lower chamber, which contained growth medium, over 24 h. Non-migratory cells were removed using a cotton swab and the transmigrated cells at the backside of the filter were stained with Giemsa (Wuhan Boster Biological Technology, Ltd.). The cells in the filter were counted under a microscope (magnification, ×400; BX53; Olympus Corp.). The data were normalized and expressed as the migration rate of the cells compared with the blank control.

### In vitro invasion assay

The Boyden chamber technique was performed to examine *in vitro* invasion. Briefly, the 8-μm pore filters were coated with 50 μl 8 mg/ml reconstituted basement membrane substance (Matrigel). The coated filters were air-dried at 4°C prior to addition of the cells. RPMI-1640 medium (600 μl) containing 5% FBS was added to the lower chambers. The cells were digested with trypsin (Wuhan Boster Biological Technology, Ltd.) and the cell suspensions (1×10^5^ cells/well) were added to the upper chambers to enable invasion for 24 h at 37°C in a humidified atmosphere of 5% CO_2_. The cells remaining attached to the upper surface of the filters were removed using cotton swabs. The migrated cells were stained with Giemsa and examined using optical microscopy (magnification, ×400). A total of five fields of view at the center and in the surrounding areas were counted and the average was calculated (19). The experiment was repeated three times.

### Data and statistical analysis

All values are expressed as the mean ± standard deviation. Statistical analysis was performed using SPSS 16.0 software (SPSS, Inc., Chicago, IL, USA). Differences among the groups were assessed using one-way analysis of variance. P<0.05 was considered to indicate a statistically significant difference between values.

## Results

### Knockdown of ADAM10 in TCA8113 cells

**Figure 1:**
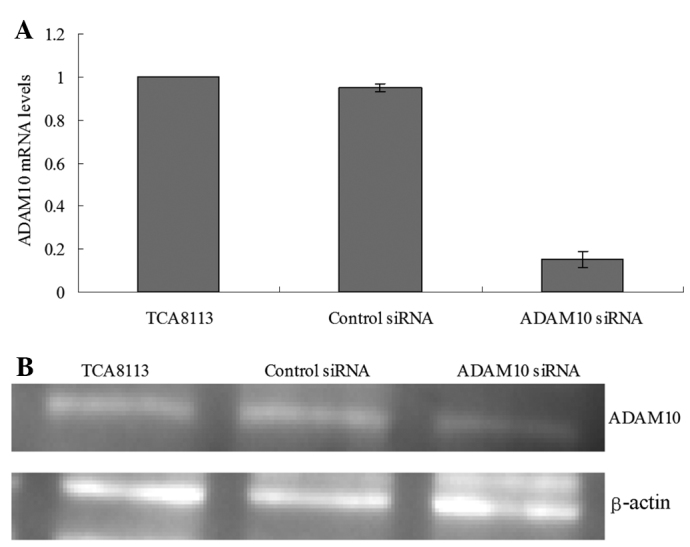
Knockdown of ADAM10 expression in TCA8113 cells 24 h after siRNA transfection. (A) ADAM10 mRNA levels were determined by reverse transcription quantitative polymerase chain reaction. Relative fold induction for ADAM10 mRNA (mean ± standard deviation) in the mock and ADAM10 siRNA-transfected TCA8113 cells is presented relative to the expression in the parental TCA8113 cells. ^*^P<0.05 compared with the parental TCA8113 cells. (B) Western blot analysis of ADAM10 protein expression in the indicated cell lines. β-actin was used as a loading control. Parental TCA8113 cells, control siRNA (mock-transfected control) and ADAM10-siRNA represent the three clones, respectively. ADAM10, A disintegrin and metalloproteinase 10; siRNA, small interfering RNA.

The expression of ADAM10 was examined using RT-qPCR and western blot analysis to validate the silencing efficiency of the target gene following RNA interference. Stably ADAM10 siRNA-transfected TCA8113 cells (ADAM10-siRNA) and a mock-transfected control cell line (control siRNA) were established, as described above. Compared with the parental TCA8113 cells and the control siRNA cells, ADAM10 mRNA and protein expression was significantly reduced in the ADAM10 siRNA cells 24 h following siRNA transfection (P<0.05; Fig. 1A and B), which persisted for at least 96 h (data not shown).

### Gene silencing of ADAM10 reduces cell proliferation and cell colony formation in TCA8113 cells

**Figure 2:**
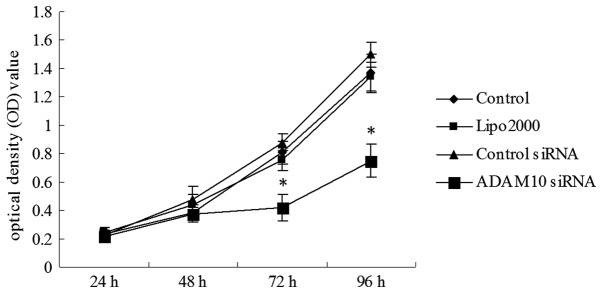
Gene silencing of ADAM10 reduces cell proliferation in TCA8113 cells. Cell proliferation was analyzed using an MTT assay. Cells were monitored for 96 h and the average optical density at 490 nm ± standard deviation for each cell line is shown. Cells transfected with ADAM10 siRNA (ADAM10-siRNA) demonstrated reduced cell growth compared with that of the blank control, Lipo2000 and control siRNA group cells 72 and 96 h after treatment, respectively. ^*^P<0.05 compared with the blank control, Lipo2000 and control siRNA group, respectively. ADAM10, A disintegrin and metalloproteinase 10; siRNA, small interfering RNA; Lipo2000, cells treated with Lipofectamine 2000.

**Figure 3:**
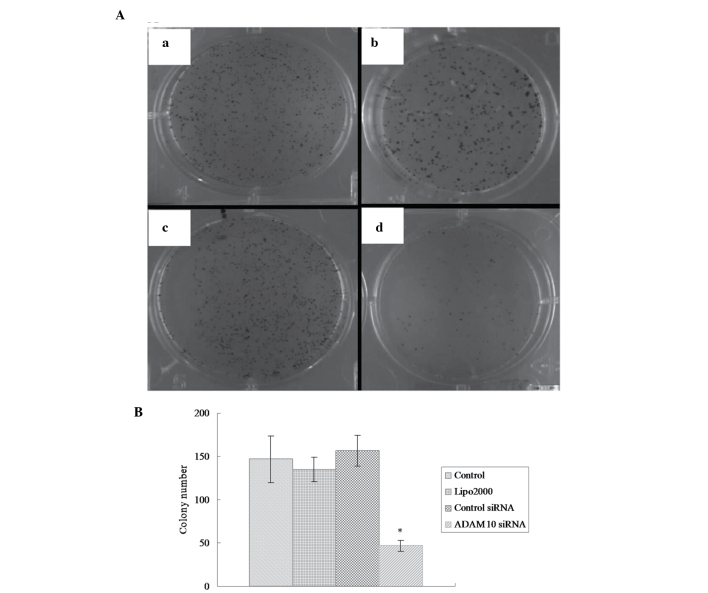
Gene silencing of ADAM10 reduces cell colony formation in TCA8113 cells. (A) ADAM10 knockdown by siRNA reduced cell colony formation (magnification, ×40), as demonstrated by the colony formation in dishes containing (a) cells in the blank control group, (b) Lipo2000 group (c) control siRNA group and (d) ADAM10-siRNA group. (B) Mean numbers of colonies of three independent experiments (mean ± standard deviation) are indicated by the histograms. ^*^P<0.05 compared with the blank control group, Lipo2000 group and control siRNA group, respectively. ADAM10, A disintegrin and metalloproteinase 10; siRNA, small interfering RNA; Lipo2000, cells treated with Lipofectamine 2000.

To examine whether the knockdown of ADAM10 expression affected cell growth, an MTT cell proliferation assay was performed. Compared with the blank control, Lipo2000 and control siRNA group cells, a decrease in cell proliferation in the ADAM10-siRNA group was observed. These results suggested that ADAM10 promoted TCA8113 cell growth (P<0.05; Fig. 2). In addition, the soft agar assay revealed that the cell colony number significantly decreased in the ADAM10-siRNA group compared with that in the blank control group, Lipo2000 group and control siRNA group (P<0.05, Fig. 3A and B).

### Gene silencing of ADAM10 reduces cell migration in TCA8113 cells

**Figure 4:**
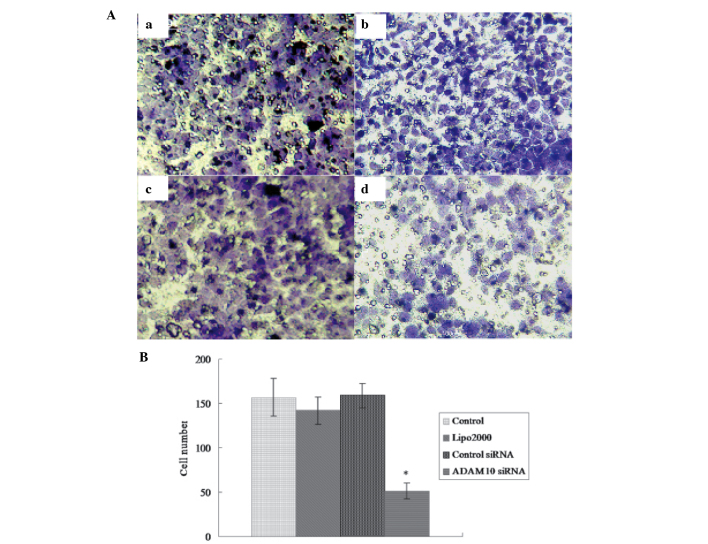
Gene silencing of ADAM10 reduces cell migration in TCA8113 cells. (A) A Matrigel Transwell invasion assay was used to assess the migration ability of cells (magnification, ×400) in the (Aa) blank control group, (Ab) Lipo2000 group, (Ac) control siRNA group and (Ad) ADAM10-siRNA group to pass through the basement membrane. (B) Values represent the cell number/field of view (mean ± standard deviation). ^*^P<0.05 compared with the blank control group, Lipo2000 group and control siRNA group, respectively. ADAM10, A disintegrin and metalloproteinase 10; siRNA, small interfering RNA; Lipo2000, cells treated with Lipofectamine 2000.

The effect of ADAM10 gene silencing on the migration of TCA8113 cells was investigated using a Transwell invasion assay (Fig. 4A). The results demonstrated that transfecting cells with ADAM10 siRNA led to a marked reduction in the capability of cells to pass through the basement membrane, compared with that of the other groups (all P<0.05; Fig. 4B). These results suggested that the expression of ADAM10 may be associated with cell migration.

### Gene silencing of ADAM10 reduces the invasive ability of TCA8113 cells

**Figure 5:**
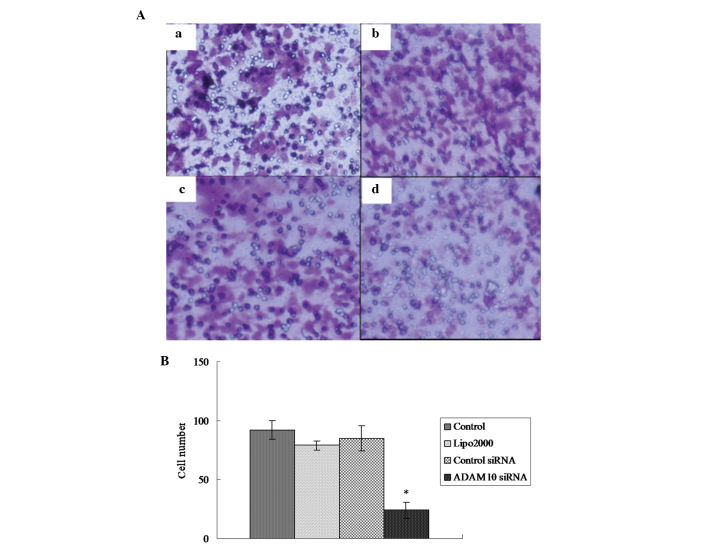
Gene silencing of ADAM10 reduces the invasive ability of TCA8113 cells. (A) A Matrigel Transwell invasion assay was used to assess the ability of TCA8113 cells to invade the filter membrane (magnification, ×400) (a) blank control group; (b) Lipo2000 group; (c) control siRNA group and (d) ADAM10-siRNA group. (B) Values represent the cell number/field of view (mean ± standard deviation). ^*^P<0.05 compared with the blank control group, Lipo2000 group and control siRNA group, respectively. ADAM10, A disintegrin and metalloproteinase 10; siRNA, small interfering RNA; Lipo2000, cells treated with Lipofectamine 2000.

A Matrigel invasion assay was used to determine the invasive potential of TCA8113 cells transfected with ADAM10 siRNA. The assay results demonstrated that the capability of the treated TCA8113 cells to pass through the basement membrane decreased markedly compared with that of the other groups (all P<0.05; Fig. 5A and B). These results indicated the importance of ADAM10 in oral cancer cell invasion.

### Silencing of ADAM10 by siRNA stimulates the activation of E-cadherin and suppresses the activation of EGFR in TCA8113 cells

**Figure 6:**
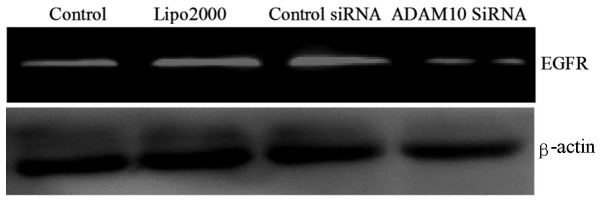
EGFR expression in TCA8113 cells 72 h after ADAM10 siRNA transfection. Western blot analysis of EGFR protein expression was performed in the indicated cell lines. β-actin was used as a loading control. Control (parental TCA8113 cells), Lipo2000 group cells, control siRNA (mock-transfected) and ADAM10-siRNA represent the different clones, respectively. ADAM10, A disintegrin and metalloproteinase 10; EGFR, epidermal growth factor receptor; siRNA, small interfering RNA.

**Figure 7:**
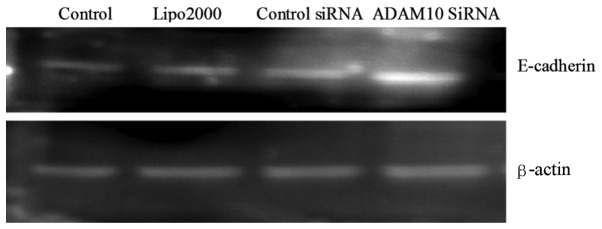
E-cadherin expression in TCA8113 cells 72 h after ADAM10 siRNA transfection. Western blot analysis of E-cadherin protein expression was performed in the indicated cell lines. β-actin was used as a loading control. Control (parental TCA8113 cells), Lipo2000 group cells, control siRNA (mock-transfected) and ADAM10-siRNA represent the different clones, respectively. ADAM10, A disintegrin and metalloproteinase 10; siRNA, small interfering RNA.

In the present study, changes in the protein levels of EGFR and E-cadherin were detected by western blot analysis. The results revealed that at 72 h after transfection, the protein levels of EGFR were significantly decreased in the TCA8113 cells treated with ADAM10 siRNA compared with those in the other groups (all P<0.05; Fig. 6), while levels of E-cadherin were significantly increased in the ADAM10 siRNA-treated cells compared with those in the cells in the other groups (all P<0.05; Fig. 7).

## Discussion

Various members of the ADAM family, including ADAM10, are overexpressed in different types of malignant tumor and may be associated with the biological behavior of the latter (9–11). The expression of ADAM10 is significantly increased in non-small cell lung cancer (NSCLC) tissues, particularly in metastatic tissues (12). Downregulation of the expression of ADAM10 with short hairpin RNA against ADAM10 has been shown to inhibit the migration and invasion of NSCLC cells (12). The present study hypothesized that the downregulation of ADAM10 may affect the biological behavior of TCA8113 cells. However, as there are few previous studies associated with this hypothesis, the purpose of the present study was to analyze the association between the gene silencing of ADAM10 and the proliferation, invasion and migration capability of TCA8113 cells *in vitro.* In addition, the association between the expression of ADAM10 and the expression of EGFR and E-cadherin proteins in TCA8113 cells was examined.

The results of the present study demonstrated that downregulation of ADAM10 resulted in the suppression of TCA8113-cell proliferation and a significant reduction in cellular invasion and migration, which indicated that ADAM10 was involved in processes of tumor development and metastasis. These findings were consistent with previous studies on the expression and functional roles of ADAM10 (9,18,20). Armanious *et al* (20) demonstrated that the active/mature form of ADAM10 is expressed in mantle cell lymphoma (MCL) cell lines and was observed in all 12 patient samples examined. The MCL cells transfected with ADAM10 siRNA demonstrated growth inhibition and cell-cycle arrest, while addition of recombinant ADAM10 to MCL cells induced a significant increase in cell growth. Yuan *et al* (9) revealed that the siRNA-mediated knockdown of ADAM10 significantly inhibited the growth, migration and invasion of HepG2 cells. Lee *et al* (18) demonstrated that the expression of ADAM10 increased markedly in melanoma metastasis compared with that in primary lesions. Downregulation of ADAM10 with siRNA led to a decrease in the growth and migration of melanoma cells. In addition, the migration of melanoma cells was induced by overexpression of ADAM10.

The effect of ADAM10 on the biological behaviors of tumor cells may be associated with its protease activity. The biological behavior of cancer cells is regulated by multiple growth factors and cytokines, a number of which are present in a membrane-bound form and which undergo proteolytic shedding in order to be activated (21). ADAM10-mediated proteolytic cleavage of substrate proteins is considered to be involved in the pathophysiology of multiple life-threatening diseases, including cancer (7). Important substrates of ADAM proteases include growth factors, cytokines and their receptors and adhesion proteins (22).

EGFR is a member of the epidermal growth factor family of receptors, a subfamily which comprises four receptor tyrosine kinases which are closely associated (23). Activation of EGFR leads to signal transduction cascades that promote cell proliferation and cell growth (23). Yan *et al* (24) demonstrated that ADAM10 stimulated the G protein-coupled receptor (GPCR) transactivation of EGFR. The effect of ADAM10 on EGFR transactivation depends on its metalloprotease activity and is due to the activation of the EGFR ligand heparin-binding EGF by GPCR signaling. In the present study, the expression of EGFR was significantly downregulated in the ADAM10 siRNA-treated cells compared with that in the control group. This indicated a positive correlation between ADAM10 and EGFR and suggested that ADAM10 may regulate the proliferation, migration and invasion of TCA8113 cells via modulating the EGFR signaling pathway.

ADAM10 may also promote tumor invasion and metastasis by degrading the extracellular matrix and affecting cell-cell signaling (25–27). Destruction of the basement membranes has been implicated as an early event in tumor metastasis. Millichip *et al* (25) revealed that cleavage of the basement membrane type IV collagen resulted from bovine ADAM10. Pan *et al* (26) demonstrated that, in the pituitary adenoma cell line AtT-20, ADAM10 facilitated cell migration via affecting the cleavage of CD44 and L1. E-cadherin is a transmembrane molecule which functions as an adhesion molecule. Increased expression of ADAM10 may lead to elevated shedding of E-cadherin and loss of cell-cell contact (18). Solanas *et al* (27) demonstrated that in epithelial cells, ephrin (Eph) B receptors interact with E-cadherin and ADAM10 at sites of adhesion and Eph/ephrin activation induces E-cadherin shedding by ADAM10. In the present study, the expression of E-cadherin was significantly upregulated in the ADAM10 siRNA-treated cells compared with that in the control group, which indicated a negative correlation between ADAM10 and E-cadherin and suggested that ADAM10 may promote the proliferation, migration and invasion of TCA8113 cells through modulation of cell adhesion and cell contact.

In conclusion, the cellular proliferation, migration and invasion abilities of the ADAM10 siRNA-transfected TCA8113 cells were significantly decreased, indicating that ADAM10 may be involved in the genesis and progression of oral cancer. Therefore, ADAM10 may serve as a potential marker and therapeutic target in the treatment of oral cancer. Activating the EGFR signaling pathway and modulating cell-cell interactions via inducing the shedding of E-cadherin may contribute to the mechanisms of action of ADAM10. Future studies are required to further investigate the molecular mechanisms underlying the involvement of ADAM10 in tumorigenesis and progression of human oral cancer cells.
